# Cohabitation is associated with a greater resemblance in gut microbiota which can impact cardiometabolic and inflammatory risk

**DOI:** 10.1186/s12866-019-1602-8

**Published:** 2019-10-22

**Authors:** Casey T. Finnicum, Jeffrey J. Beck, Conor V. Dolan, Christel Davis, Gonneke Willemsen, Erik A. Ehli, Dorret I. Boomsma, Gareth E. Davies, Eco J. C. de Geus

**Affiliations:** 1 Avera Institute for Human Genetics, Avera McKennan Hospital & University Health Center, Sioux Falls, SD USA; 2 Amsterdam Public Health Research Institute, Vrije Universiteit Medical Center, Amsterdam, The Netherlands; 3 Department of Biological Psychology, Behavioral and Movement Sciences, Vrije Universiteit, Amsterdam, The Netherlands

**Keywords:** Gut microbiota, Cohabitation, Twin genetics

## Abstract

**Background:**

The gut microbiota composition is known to be influenced by a myriad of factors including the host genetic profile and a number of environmental influences. Here, we focus on the environmental influence of cohabitation on the gut microbiota as well as whether these environmentally influenced microorganisms are associated with cardiometabolic and inflammatory burden. We perform this by investigating the gut microbiota composition of various groups of related individuals including cohabitating monozygotic (MZ) twin pairs, non-cohabitating MZ twin pairs and spouse pairs.

**Results:**

A stronger correlation between alpha diversity was found in cohabitating MZ twins (45 pairs, r = 0.64, *p* = 2.21 × 10^− 06^) than in non-cohabitating MZ twin pairs (121 pairs, r = 0.42, *p* = 1.35 × 10^− 06^). Although the correlation of alpha diversity did not attain significance between spouse pairs (42 pairs, r = 0.23, *p* = 0.15), the correlation was still higher than those in the 209 unrelated pairs (r = − 0.015, *p* = 0.832). Bray-Curtis (BC) dissimilarity metrics showed cohabitating MZ twin pairs had the most similar gut microbiota communities which were more similar than the BC values of non-cohabitating MZ twins (empirical *p*-value = 0.0103), cohabitating spouses (empirical p-value = 0.0194), and pairs of unrelated non-cohabitating individuals (empirical p-value< 0.00001). There was also a significant difference between the BC measures from the spouse pairs and those from the unrelated non-cohabitating individuals (empirical *p*-value< 0.00001). Intraclass correlation coefficients were calculated between the various groups of interest and the results indicate the presence of OTUs with an environmental influence and one OTU that appeared to demonstrate genetic influences. One of the OTUs (Otu0190) was observed to have a significant association with both the cardiometabolic and inflammatory burden scores (p’s < 0.05).

**Conclusions:**

Through the comparison of the microbiota contents of MZ twins with varying cohabitation status and spousal pairs, we showed evidence of environmentally influenced OTUs, one of which had a significant association with cardiometabolic and inflammatory burden scores.

## Background

The gut microbiome plays an important role in health and disease, in a wide range of areas spanning outcomes in the cardiometabolic, immune and mental health domains [1–3]. The composition of the gut microbiota is now understood to be influenced by a number of factors including the host genetic profile [4, 5] and a myriad of environmental influences. These environmental factors can include the seeding at birth [6, 7], the composition of mothers milk or formula [8], exposure to pathogens [9] and health-associated behaviors like dietary intake and exercise activity [10–12]. Human studies comparing the microbiome of family members have been important in delineating the role of these various factors in influencing the human gut microbiome. Similarities in the gut microbiome of family members living together may be due to shared genetic factors, shared past, or current environmental exposures. Shared environmental factors may range from a shared womb (as applies to siblings and especially twins) passage through the birth canal to shared parenting rearing styles (breastfeeding), as well as all other exposures resulting from the actual sharing of a household (e.g. dietary habits, pet exposure, pollutant exposure). These common exposures may extend beyond the immediate household to an exposome shared by family members that includes neighborhood characteristics. Specific knowledge of the contribution of household effects to the abundance of specific microbial taxa could help delineate interventions to influence microbiome compositions associated with specific disease burden.

By comparing the resemblance of the gut microbiota of monozygotic (MZ) and dizygotic (DZ) twins, the relative contribution of genetic factors and shared environment lending to individual variation in the microbiome can be estimated, without the need to measure the genome or the environment [4, 5, 13, 14]. However, the shared environment is mainly reflective only of shared household effects up until late adolescence, at which point twins will typically move out of the family home.

When considering familial resemblances in adulthood, individuals typically will have started their own families and started to share their environment with others to whom they are not related (e.g. their spouses) or with whom they share genes and environment (e.g. their offspring). In the classical twin design, the effects of the current household of the twins tend to become subsumed under the non-shared or unique environment, to the extent that previous experience and genotype do not influence current household environment. Unique environment may also include many other environmental factors unrelated to the home environment such as exposures at work, as well as all measurement error. Therefore, estimation of the effects of sharing a household on the adult microbiome requires a unique design which recognizes current sharing in addition to carry over effects from previous household sharing.

Previous work has demonstrated that aspects of the gut microbiome composition are influenced by the individuals within a shared household, particularly between spouse pairs [15–17]. The impact that contact with others has on microbiota composition has even been found to extend to non-family members in a shared household as well as individuals within our social networks [14, 17]. Although these studies have provided evidence that cohabitation is capable of influencing the gut microbiota, it is necessary to confirm that microbes shared amongst cohabitating individuals are truly shared due to a common environment and not aspects of shared genetic ancestry. This is the case even for studies that focus on microbiome composition similarities amongst spouses and social networks as it has been previously demonstrated that spouse pairs and individuals within a similar social group resemble each other genetically more so than unrelated individuals [18, 19].

Here we detected shared household effects by two different strategies. First, we compared young adult MZ twins who still share a household (cohabitating) with each other to adult MZ twins who no longer share a household (non-cohabitating). Both types of MZ twin pairs are genetically identical. A larger resemblance in the abundance of specific (taxa of) microbes in younger MZ twins, who still share a household, compared to older MZ twin who do not, would reflect shared household effects. By observing OTUs strongly correlated between cohabitating MZ twin pairs but not non-cohabitating MZ twin pairs, we can be confident that the identified microbes are not influenced by genetic similarities. Second, we also tested whether similar cohabitation effects were found in unrelated persons sharing a household by comparing the resemblance in the gut microbiome of spouse pairs currently sharing a household to that of randomly matched pairs of the same age who never shared a household. For completeness, and to replicate previous findings that host genetic factors contribute to variation in the microbiome [4, 20–22], we further explored the microbiome resemblance in the MZ twins who do not share a household.

To explore the clinical relevance of the microbes detected to be sensitive to shared household effects, we computed the cardiometabolic and pro-inflammatory burden profiles based on a host of fasting blood-derived parameters [23]. Under the hypothesis that shared household can influence disease burden with the microbiome as a mediator, we expect the microbes that show a significant shared household effect to be associated with different metabolic and pro-inflammatory burden.

## Methods

### Participants

Study participants consisted of individuals registered with the Netherlands Twin Register (NTR) (*N* = 419, 272 females, 147 males). The majority of individuals assayed were MZ twins and their spouses (MZ *N* = 332 (166 pairs), DZ *N* = 6 (3 pairs), spouses of MZ twins = 42, unrelated individuals = 39). Within the group of MZ twins, 45 pairs still cohabitated (mean age = 23.33, range 19-68), and 121 pairs no longer did so (mean age = 35.76, range 19-59, minimum live-apart time: 1 year, mean live-apart time: 17.77 years).

### Fecal collection, DNA isolation and sequencing

Fecal samples collected from participants were stored at 4 °C until delivered to the laboratory within a 36-h period. Anaerocult was used in order to preserve anaerobic species present within a sample. The samples were homogenized, aliquoted, and stored at − 80 °C until used for microbial DNA extraction. DNA extraction was performed using the Qiagen Powersoil kit with the addition of the heating step from the Powerfecal kit (heating at 65 °C for 10 min). Resulting microbial DNA was subjected to PCR in order to amplify the V4 region of the 16S rRNA gene. The resulting library fragments were normalized using the SequalPrep normalization plates (Invitrogen, Carlsbad, CA). The libraries were pooled and analyzed via an Agilent Bioanalyzer trace. Samples were split into two sequencing runs in order to increase sample read depth. Samples were grouped together by family groups (twins, spouses) in order to make sure all samples from a family were sequenced in the same sequencing run. Sequencing data was generated on the MiSeq platform, using a 2 × 251 paired-end sequencing run with 20% Phix to increase base diversity during the run.

### Sequence processing

Sequence processing was carried out as previously described [24, 25]. Briefly, after the DNA sequencing process, demultiplexed forward and reverse reads were obtained after the DNA sequencing process using Mothur (version 1.39.5 )[26]. Forward and reverse reads were overlapped in order to obtain contigs. We subsequently screened to discard reads longer than 275 base-pairs or reads that contained any ambiguous base calls. Unique reads were then aligned to a trimmed version of the SILVA (version 128) database containing the V4 region of the 16S rRNA gene. Reads that fell outside of this region were discarded. Performing the preclustering step, reads that only differed by up to two nucleotides were grouped. Chimera detection was performed using the VSEARCH algorithm (version 2.3.4) and probable chimeric reads were removed. Sequences were classified using a Bayesian classifier trained on the RDP database (version 16). Non-bacterial reads were removed from downstream analysis. After the aforementioned quality control process, sequence reads were clustered into species level operational taxonomic units (OTUs) at a 97% similarity cutoff through the use of the Opti-Clust algorithm [27], a de novo OTU clustering method implemented in Mothur (version 1.39.5). The formed OTUs were taxonomically labelled using the consensus taxonomy for each OTU. In order to explore higher taxonomic levels, phylotype binning was performed based on the classification of each sequence read. Phylotyping was performed at both the genus and family levels using Mothur (version 1.39.5). Total reads for each sample were subsampled to the depth of the sample with the lowest sequence coverage (16,242 reads). After subsampling, alpha diversity was calculated for each sample in the form of Shannon and Chao1 indices. Beta diversity measures were also generated by computing Bray-Curtis dissimilarity measures between all individuals. A mock community was also sequenced along with the samples. Analysis of the mock community sequences after the sequence QC process determined the error rate to be 0.00253%.

### Cardiometabolic and inflammatory factor measurement

Data on a number of cardiometabolic and inflammatory factors were available for all of participants within the study. The measurement of these factors has been previously described elsewhere as well as the criteria for exclusion based on laboratory measurements, such as the measurements falling outside the limit of detection for a particular assay [23]. Cardiometabolic measures included body mass index (BMI), waist-hip ratio (WHR), LDL-cholesterol, HDL-cholesterol, triglycerides, glucose, insulin, systolic blood pressure (SBP), and diastolic blood pressure (DBP). Inflammatory traits included fibrinogen, interleukin-6 (IL-6), C-reactive protein (CRP), and tumor necrosis factor alpha (TNF-α). These data were used to generate disease burden scores separately for both inflammatory and cardometabolic traits. Disease burden scores were standardized (i.e., mean of zero, standard deviation of one). To ensure that an increase in the variables assayed is associated with an increase in disease burden, the scale of some variables (e.g., HDL) were reversed by multiplying the standardized score by − 1. Next, following the metabolic syndrome definition of the American Heart Association, we summed Z-scores for WHR, triglycerides, HDL-cholesterol, SBP and glucose to a single cardiometabolic burden score. There were 416 individuals that had valid measurements for all of the cardio-metabolic factors utilized. An additional pro-inflammatory burden score was computed by summing the Z-scores for fibrinogen, IL-6, CRP, and TNF-α. There were 401 individuals that had valid measurements for all of the inflammatory factors utilized.

### Statistical analyses

After generation of Shannon and Chao1 indices for all participants, we sought to compare the resemblance of gut microbiota alpha diversity between individuals forming a twin or spouse pair and individuals sharing and not sharing a household. Pearson correlations in alpha diversity were computed in 1) cohabitating MZ twin pairs, 2) he non-cohabitating MZ twin pairs, 3) spouse pairs, and 4) pairs of randomly selected unrelated individuals who did not share a household. Selection of the latter pairs was performed in a manner that ensured the resulting unrelated pairs were not matched with the spouse of a co-twin and that both unrelated individuals were sequenced in the same sequencing batch. Matching a twin to the spouse of a co-twin could possibly inflate the level of similarity between the unrelated individuals whereas inclusion of unrelated pairs derived from multiple sequencing batches could artificially inflate the dissimilarity of the unrelated individuals relative to the various family pairings (MZ twins or spouse pairs), which were always sequenced in the same sequencing batch. To confirm the effects of the household on adult gut microbiota composition, Bray-Curtis (BC) dissimilarity measures based on the species OTU counts were calculated. BC measures of cohabitating and non-cohabitating twins were compared using a t-test with 10,000 permutations. Likewise, the BC measures of cohabitating spouse pairs were compared to that of non-cohabitating unrelated pairs who were sequenced in the same sequencing batch (to account for the fact that all family members – twins and spouses- were in the same sequencing batch).

Finally, restricting the our analyses to OTUs present in 40% of individuals, we computed the intraclass correlation coefficients (ICCs) in the four different sets of pairings (cohabitating MZ pairs, non-cohabitating MZ twin pairs, unrelated spousal pairs sharing a household, and unrelated opposite sex pairs not sharing a household) for *individual* species level OTUs to detect household effects on specific OTUs. OTUs were restricted to those present in 40% of the individuals to ensure to restrict the range of sample sizes used to estimate the ICC’s. Otherwise vastly different sample sizes. (i.e. ranging from 5 to 419) would cause strong sampling variation bias in the estimation of the ICCs. Unrelated opposite sex pairs were generated in a similar manner to the pairs of unrelated individuals described above with the addition of making sure the unrelated individuals were of the opposite sex and within 4 years of age. The threshold age difference was chosen because the mean difference in age between the spouses in the data was 3.4 years. We also tested the group differences in ICCs at the family, genus and species level using an F-test, with a Bonferroni correction on the subsequent *p*-values to account for multiple testing.

We considered OTUs to be affected by the shared household if they had significant intrapair similarity in cohabitating MZ pairs *and* significant intrapair similarity in spouse pairs but not in non-cohabitating MZ twin pairs or unrelated opposite sex pairs with a similar age distribution. For completeness we also identified OTUs with significant intrapair similarity in MZ pairs, whether cohabitating or non-cohabitating, and interpreted these as reflective of predominant genetic effects.

Given that mode of birth (cesarean section vs. vaginal birth) has been shown to at least influence the gut microbiota composition at least temporarily [28], we repeated all analyses after the exclusion of the individuals born via cesarean section (*N* = 43) to see how this impacted the results.

## Results

### Shared household effects on alpha diversity

**Fig. 1:**
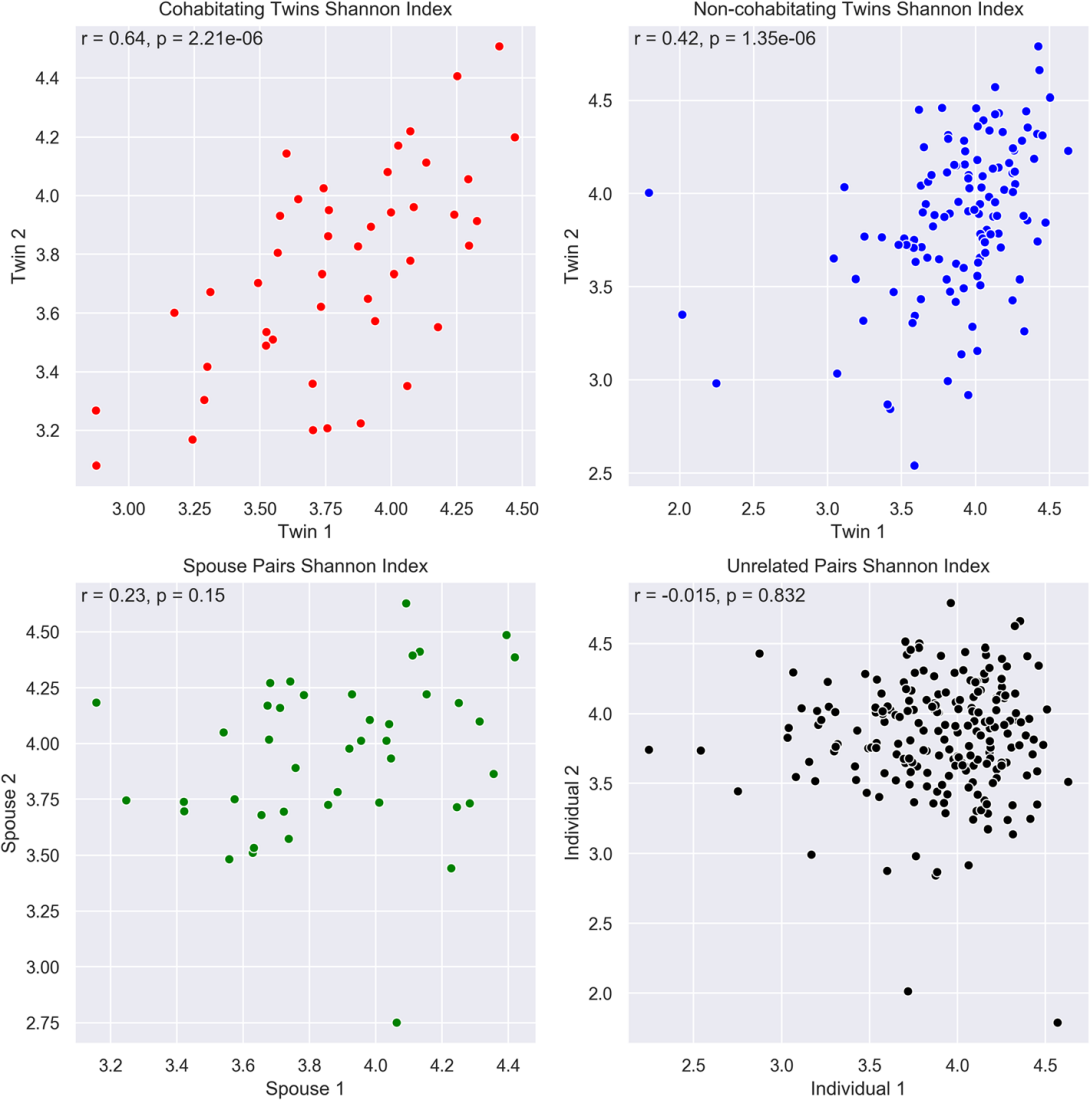
Alpha diversity correlations between the different groups of individuals with varying degrees of relatedness

Figure 1 displays scatterplots of alpha diversity in the four parings of interest. Non-cohabitating MZ twin pairs (121 pairs) showed a moderately strong correlation between alpha diversity measurements (r = 0.42, *p* = 1.35 × 10^− 06^). The cohabitating MZ twin pairs (45 pairs) showed a stronger correlation relative to the non-cohabitating twins (r = 0.64, *p* = 2.21 × 10^− 06^). The Pearson correlation of the alpha diversity in the unrelated, cohabitating spousal pairs (42 pairs) did not attain significance (r = 0.23, *p* = 0.15) but were still higher than those in the 209 unrelated pairs (r = − 0.015, *p* = 0.832). This same pattern of results generally held true with the Pearson correlations performed on the Chao1 indices (cohabitating MZ: r = 0.66, *p* = 6.77 × 10^− 07^, non-cohabitating MZ: r = 0.58, *p* = 3.26 × 10^− 12^, spouse pairs: r = 0.13, *p* = 0.40, unrelated pairs: r = − 0.046, *p* = 0.51). These comparisons were repeated after exclusion of the cesarean-born individuals. The pattern of the results did not change.

### Shared household effects reflected in beta diversity

**Fig. 2:**
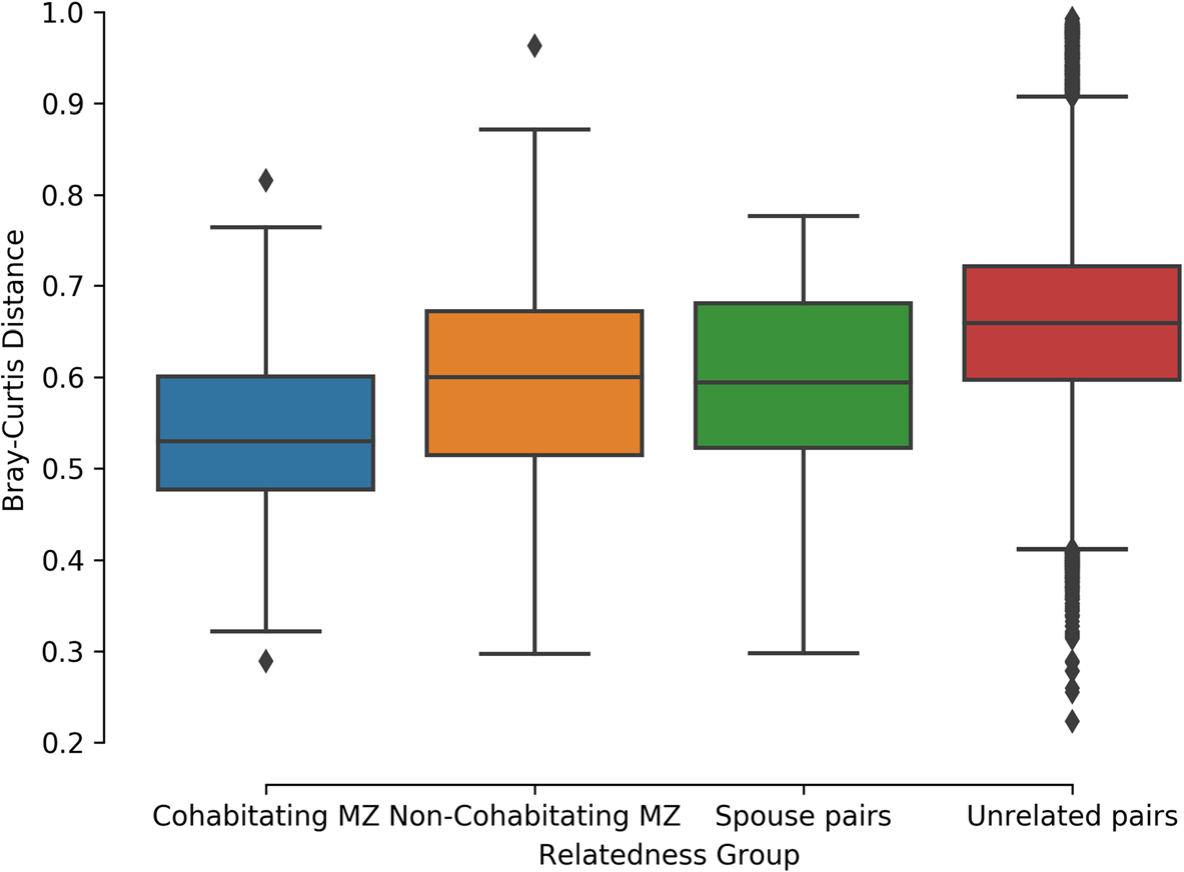
Boxplot of Bray-Curtis dissimilarity corresponding to the various relatedness groups

Bray-Curtis dissimilarity metrics were generated between all individuals in the study. Figure 2 provides a boxplot of the BC values generated using all species level OTUs. A BC dissimilarity matrix was used as input for the principal coordinate analysis for visualization purposes (Additional file 1). A series of t-tests including 10,000 permutations were used to determine differences in the mean BC metrics between any of the different groups with varying degrees of relatedness. Cohabitating MZ twin pairs had the most similar gut microbiota communities (lowest mean BC values) which was significantly lower than the BC of non-cohabitating MZ twins (empirical *p*-value = 0.0103), cohabitating spouses (empirical *p*-value = 0.0194), and pairs of unrelated non-cohabitating individuals (empirical *p*-value = < 0.00001). There was also a significant difference between the spouse pairs and the unrelated non-cohabitating individuals (empirical *p*-value = < 0.00001).

### Shared household effects on genus and family levels

Intraclass correlation coefficients were calculated between the aforementioned four sets of pairings for the genus and family taxons present in at least 40% of individuals. At the genus level there were 6 genera significantly correlated between non-cohabitating twins, 9 genera significantly shared between cohabitating MZ pairs, 3 shared genera amongst spouses and 1 genus that showed a significant correlation between the randomly generated opposite sex pairs (Table 1). The only overlapping genera was significantly correlated between cohabitating and non-cohabitating MZ pairs. This corresponded to an unclassified genus within the *Firmicutes* phylum.

**Table 1:** Genera and families identified as having a significant intraclass correlation coefficient (Bonferroni corrected *p*-value < 0.05)

	Cohabitating MZ	Non-Cohabitating MZ	Spouse
Genus	Firmicutes_unclassified*	Firmicutes_unclassified*	Barnesiella
	Senegalimassilia	Intestinimonas	Porphyromonadaceae_unclassified
	Bacteria_unclassified	Dialister	Paraprevotella
	Veillonella	Akkermansia	
	Romboutsia	Terrisporobacter	
	Olsenella	Anaerostipes	
	Enterobacteriaceae_unclassified		
	Erysipelotrichaceae_unclassified		
	Flavonifractor		
Family	Firmicutes_unclassified*	Firmicutes_unclassified*	Firmicutes_unclassified*
	Bacteria_unclassified	Verrucomicrobiaceae	Porphyromonadaceae
	Enterobacteriaceae	Peptostreptococcaceae	
	Peptostreptococcaceae	Ruminococcaceae	
	Ruminococcaceae		

* indicates same unclassified taxon

At the family level there were 5 significantly correlated taxonomic families between cohabitating MZ pairs, 4 between non-cohabitating MZ pairs, 2 shared between spouses and no significantly correlated families between the randomly generated, opposite sex pairs. The family level showed greater overlap of the microorganisms shared between the sets of cohabitating and non-cohabitating MZ pairs with 3 families significantly correlated between both sets of MZ twins (Table 1). One of these families, an unclassified family within the *Firmicutes* phylum, was also significantly correlated between spouse pairs.

### Shared household effects on species level microbes

**Fig. 3:**
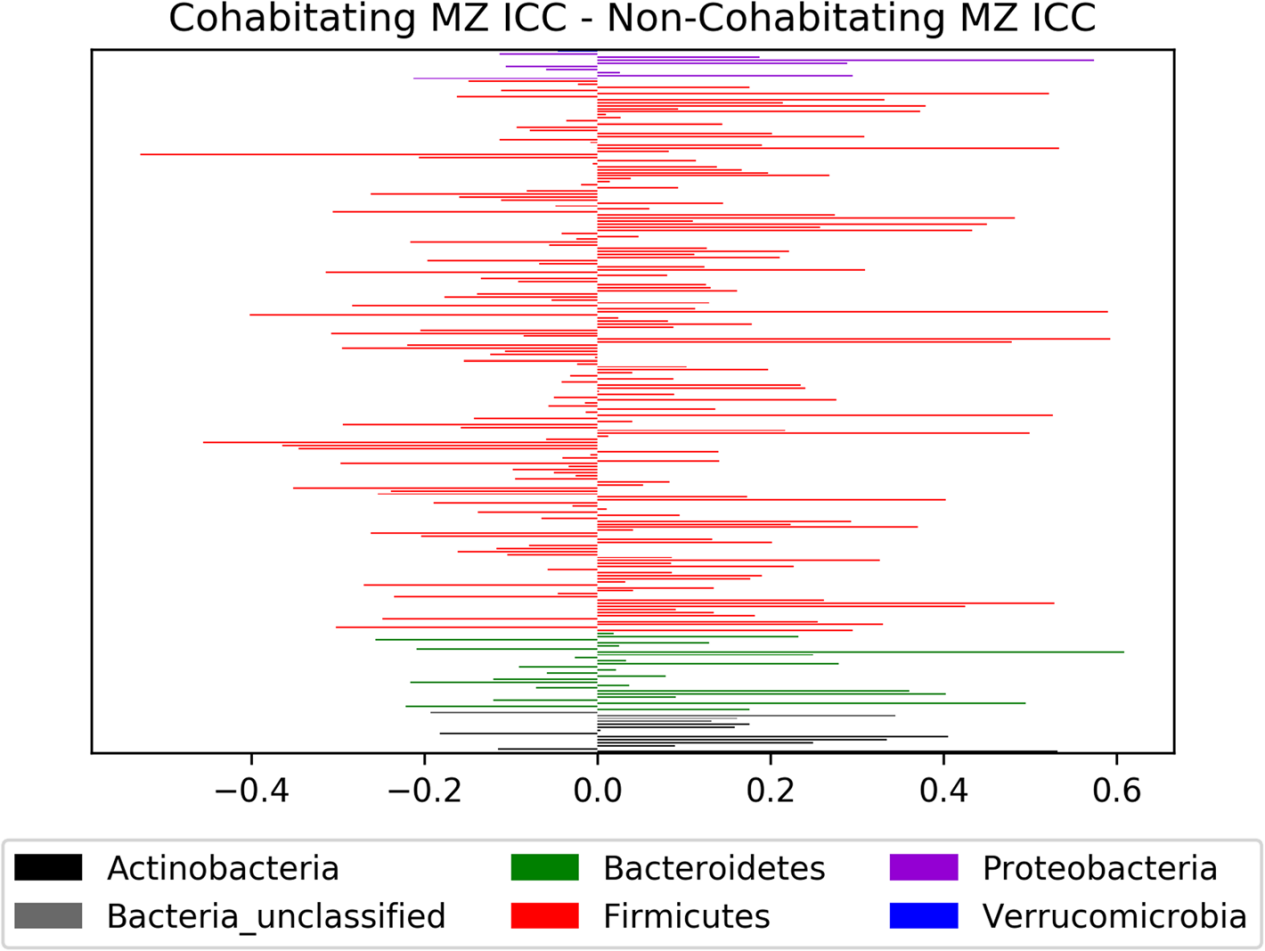
Difference in the intraclass correlation coefficients (ICC) from the cohabitating and non-cohabitating twin pairs. Bars are labeled with the phylum classification of the species level OTU (0.03 cutoff). Bars that extend to the left indicate a larger intraclass correlation coefficient in the non-cohabitating MZ pairs for that particular OTU (Non-cohab. ICC > Cohab. ICC), whereas bars extending to the right indicate a larger intraclass correlation coefficient in the cohabitating MZ pairs relative to the non-cohabitating MZ pairs (Cohab. ICC > Non-cohab.)

**Fig. 4:**
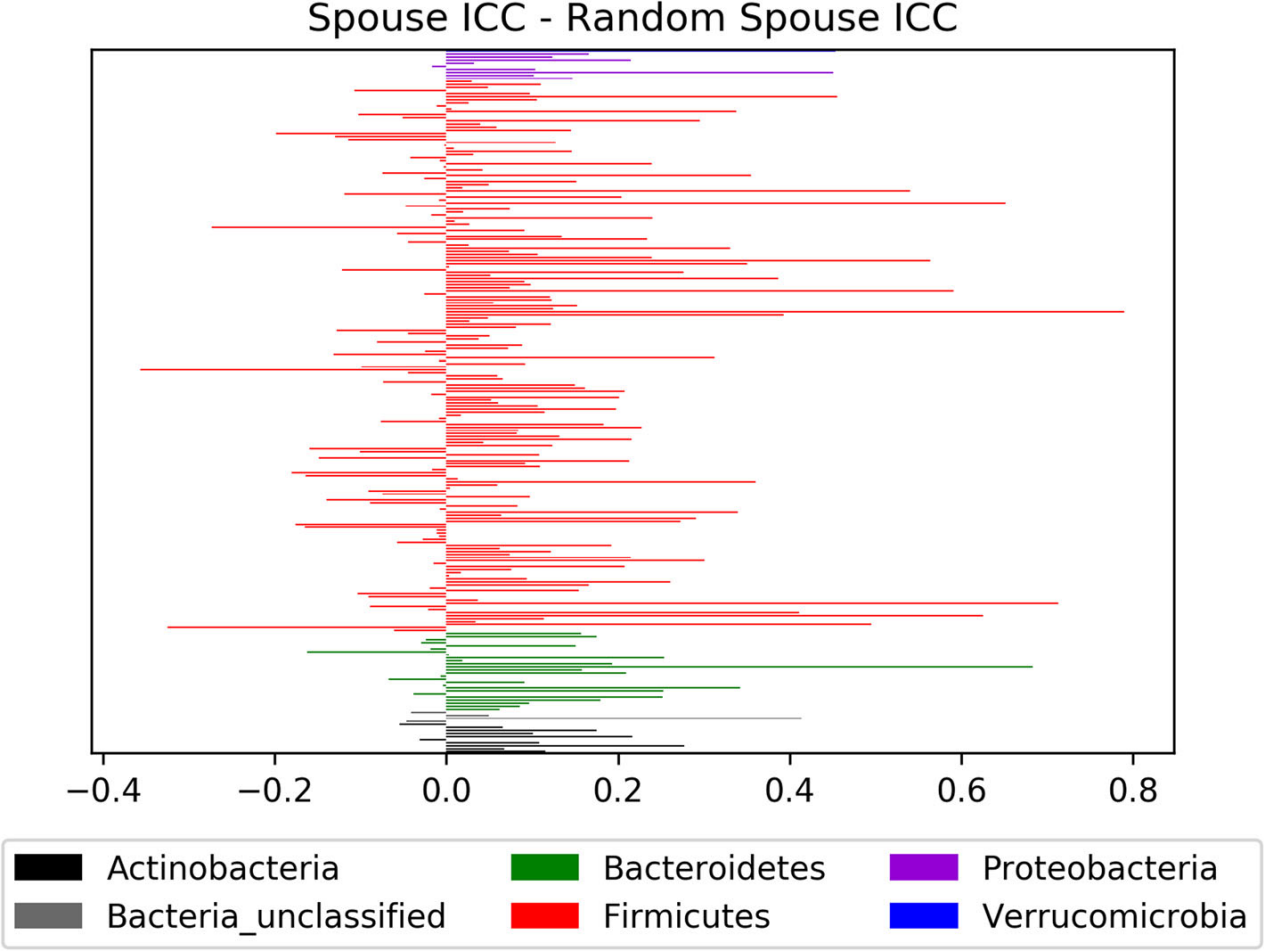
Difference in the intraclass correlation coefficients (ICC) from the spouse pairs and the randomly generated spouse pairs. Bars are labeled with the phylum classification of the species level OTU (0.03 cutoff). Bars that extend to the left indicate a larger intraclass correlation coefficient in the unrelated spouse pairs for that particular OTU, whereas bars extending to the right indicate a larger intraclass correlation coefficient in the actual pairs relative to the unrelated spouse pairs

Figure 3 shows that the ICC for cohabitating MZ pairs at the species level was generally higher than that for non-cohabitating pairs across all analyzed species level species level OTUs. Figure 4 similarly presents the difference between the intraclass correlation coefficients of the spouse pairs and the random opposite sex pairs. Species level OTUs were then separated based upon the consensus phylum classification of the OTU to determine the proportion of OTUs within a phylum that have a higher ICC in cohabitating MZ twins relative to non-cohabitating MZ twins (Table 2).

**Table 2:** Percentage of species level OTUs within a phylum that have a greater ICC value in the cohabitating MZ twins relative to the non-cohabitating twins. Phylum classification was based on the consensus taxonomic classification of the OTU

Phylum of OTU	# of OTUs	PERCENTAGe Cohab ICC > Non-Cohab ICC
Actinobacteria	10	80%
Unclassified bacteria	4	75%
Bacteroidetes	26	61.5%
Firmicutes	182	54.4%
Proteobacteria	9	55.6%
Verrucomicrobia	1	0%

Maintaining strict correction for multiple testing (928 comparisons), the cohabitating MZ twin pairs had 13 OTUs with a corrected p-value meeting the predefined cutoff of 0.05, and the non-cohabitating MZ twin pairs also had 13 significant OTUs. Otu0095 was the only significant OTU that overlapped between the cohabitating (Otu0095 ICC: 0.625, F-stat: 4.34, *p*-value: 0.0013) and non-cohabitating MZ twins (Otu0095 ICC: 0.666, F-stat: 2.08, *p*-value: 0.032), suggesting a predominant genetic influence on intrapair resemblance.

ICC calculations between cohabitating spouse pairs resulted in 4 significant OTUs. In contrast, none of the randomly matched non-cohabitating pairs of random opposite sex pairs had significant OTUs. Of particular interest are Otu0081 and Otu0190 because these two species level OTUs showed a particularly strong relationship between both cohabitating MZ twin pairs (Otu0081 ICC: 0.676, F-stat: 5.18, *p*-value: 9.23 × 10^− 5^; Otu0190 ICC: 0.660, F-stat: 4.87, p-value: 2.31 × 10^− 4^) as well as the spousal pairs (Otu0081 ICC: 0.762, F-stat: 7.42, p-value: 9.26 × 10^− 7^; Otu0190 ICC: 0.666, F-stat: 5.00, p-value: 3.96 × 10^− 4^) but not in non-cohabitating MZ twin pairs.

### Association of the shared household microbiome effects with cardiometabolic and inflammatory burden scores

Since the shared household affects alpha diversity we first tested whether, across all participants, alpha diversity was associated with either cardiometabolic or inflammatory burden profiles, which did not yield significant results at a predefined alpha of 0.05.

Next, the species level OTUs previously observed as being particularly modulated by household effects (Otu0081 and Otu0190) were further explored in order to determine whether either of these OTUs were associated with cardiometabolic or inflammatory burden profiles. Burden scores were regressed on the OTU counts in the full sample using a Generalized Estimating Equation (GEE) regression accounting for the relatedness of the MZ twins. Age and sex were also included in the GEE models. Otu0190 was observed to have a significant association with both the cardiometabolic and inflammatory burden scores (beta = − 0.0072, *p* < 0.05; beta = − 0.0085, p < 0.05, respectively). Otu0081 was not significantly associated with either the inflammatory or cardiometabolic burden scores.

Because cohabitating twins tend to be younger than non-cohabitating twins, our design assumes that there are no systematic age effects on intrapair resemblance confounding the comparison of habituating versuis non-habituating resemblances. We tested this explicitly by regressing intrapair differences for the OTUs of interest on age, household status and the interaction of age and household status. We did not observe any significant associations between the predictors and the intrapair resemblance at a predefined alpha of 0.05.

## Discussion

Our results highlight a role for shared household effects on the adult gut microbiome. This held strongly for alpha diversity and beta diversity measures, with household effects on family, genus, or species levels harder to pinpoint. However, the species level OTUs, Otu190 and Otu0081, found to be significantly associated between cohabitating twin pairs and spouse pairs, but not within the non-cohabitating MZ twin pairs, provide direct evidence that specific members of the gut microbiota can be heavily influenced by environmental conditions. These findings support previous work that showed that beta diversity values as well as bacterial SNP variant similarity were positively correlated with the number of years that 32 MZ and 92 DZ twin pairs lived apart [5]. A number of other studies have also consistently demonstrated that twin pairs are more similar in gut microbiome composition from early life [29] as well as later in life [4] with lower beta-diversity measurements between twin pairs relative to unrelated individuals. Results are also congruent with the significant similarities in the microbiomes found in 32 genetically unrelated individuals, who reported sharing a household and subsequently microbiome composition at the species level [14].

While alpha diversity appears to show a trend towards a household effect, it is worth noting the fairly strong correlation in alpha diversity measurements between MZ twins that are no longer cohabitating. This demonstrates that the shared household effect on gut microbiota alpha diversity is either so pronounced that similarities are still observed for long periods after cohabitation, or there are additional genetic effects on alpha diversity. Previous work performed in twin based microbiome studies has shown that the gut microbiome alpha diversity has a weak heritable component [21], while another recent study did not find evidence of similarities in alpha diversity between individuals of similar ancestry or genetic kinship [14]. Taken together, current evidence suggests a strong role for environmental influences on the gut microbiota composition.

Earlier studies in family members have often used the ongoing sharing of a household by spouses or siblings to detect its effects on the microbiome. However, these designs does not take into account however that the resemblance of spouses can be decreased through sex effects on the microbiome as spouse pairs are (in majority) of opposite sex. Comparing resemblances in siblings or parent-offspring designs will confound the shared household effects with shared genetic effects on the microbiome, which are known to be non-zero [4, 21]. The classical twin design can estimate the relative contribution of genetic factors and shared environment to variation in the microbiome but then the shared environment is not-specific for the actual current sharing of a household and includes sharing of pre- and perinatal and early life factors. By comparing adult MZ twin pairs that do or do not share a household, where both groups contain genetically identical individuals but differ in living status (whether living with twin or spouse), we obtain the least confounded view on the association of the current household with the microbiome composition. Our results show a reassuring convergence of the various designs. The systematic presence of more sharing of microorganisms between cohabitating compared to non-cohabitating MZ twin pairs *and* between spouse pairs compared to random male-female pairs and demonstrates the ability of a shared household to modulate specific microbiota members regardless of genetic similarity or sex.

Otu190 and Otu0081 were given consensus classifications as *Ruminococcaceae* and *Clostridiales* respectively. We attempted to further taxonomically characterize these species level OTUs by generating sequences that best represent these OTUs and performing a BLAST search with the subsequent FASTA file. OTU81 returned results indicating an uncultured *Oscillibacter* species, which resides within the *Clostridales* order. OTU190 returned more ambiguous results, which were largely classified as uncultured bacterium with an occasional hit reaffirming the consensus taxonomic classification of *Ruminococcaceae*. It should be noted that because the OTUs are derived from de novo OTU clustering of pairwise sequence distances, the consensus classification process results in varying levels of classification with Otu0081 classified to the family level and Otu190 classified to the order level. In fact, *Ruminococcaceae* is a member of the *Clostridiales* order. Organisms belonging to the *Ruminococcaceae* family have been shown in previous studies to be impacted by both high fat diet and exercise activity [11]. A shared diet is an obvious component that may account for the found shared household effects here, both with regard to the alpha and beta diversity measures as the specific effects on the *Clostridiales* OTUs. Some evidence for clinical relevance of this shared household effects was found in the downstream effects of Otu0190 on both the inflammatory burden score and the cardiometabolic burden score.

Otu0095 showed a very strong resemblance within MZ pairs, independent of whether they were cohabitating or non-cohabitating, suggesting a predominant genetic influence on the intrapair resemblance for this OTU. Consensus classification of Otu0095 determined the OTU belonged to the *Lachnospiraceae* family. Previous work by Goodrich et al. [4] determined that the amounts of *Lachnospiraceae* were more similar between MZ twins relative to DZ twins. Furthermore, *Lachnospiraceae* was identified as one of the two taxonomic families that contained the majority of OTUs with the highest heritability estimate. We did not observe evidence of this OTU influencing either the cardiometabolic or inflammatory disease burden scores.

By exploiting the rapid progress in molecular genetic technology we have excellent strategies at our disposal to identify the elements in the microbiome that are influenced by genetic factors, and large scale international efforts for genome wide association studies are underway [30]. To detect environmental influences, including those of a shared household, the main strategy has been to select and measure a specific environmental factor and test its covariance with microbiome composition. A disadvantage of that strategy is that we do not uncover the effects of yet unknown environmental factors. The approach employed here, comparing cohabitating and non-cohabitating twins, and cohabitating spouse pairs to age-matched non-cohabitating pairs, provides a route to test the effects of unmeasured environmental factors on the microbiome, at least with regard to the shared household component.

## Conclusions

Through the sampling of cohabiting MZ twins, non-cohabitating MZ twins and spouse pairs, we were afforded the unique opportunity to observe similarities and differences between these groups with regard to the gut microbiota, highlighting a role of cohabitation in shaping the gut microbiota composition. This study clearly demonstrates that cohabitation results in a similar gut microbiota alpha diversity and lower BC distances relative to unrelated individuals. Furthermore, individual OTUs at varying taxonomic levels were found to be impacted by a shared household status. One species level OTU was found to be significantly associated with both cardiometabolic and inflammatory disease burden.

## Supplementary information

**Additional file 1: Fig. S1.** PCoA plot generated from a Bray-Curtis dissimilarity matrix for visualization purposes.

## Data Availability

The Netherlands Twin Register has a data access committee that reviews data requests and will make data available to interested researchers. The data come from extended twin families and pedigree structures with twins, which create privacy concerns. Researchers may contact prof Eco de Geus (eco.de.geus@vu.nl).

## Abbreviations

BC: Bray-Curtis
BMI: Body Mass Index
CRP: C-Reactive Protein
DBP: Diastolic Blood Pressure
DZ: Dizygotic
GEE: Generalized Estimating Eqs.
HDL: High Density Lipoprotein
ICC: Intraclass Correlation Coefficient
IL-6: Interleukin 6
LDL: Low Density Lipoprotein
MZ: Monozygotic
NTR: Netherlands Twin Register
OTU: Operational Taxonomic Unit
SBP: Systolic Blood Pressure
SNP: Single Nucleotide Polymorphism
TNF-α: Tumor Necrosis Factor α
WHR: Waist-Hip Ratio
