# Supplementary figures and images for: Cohabitation is associated with a greater resemblance in gut microbiota which can impact cardiometabolic and inflammatory risk
Source: BMC Microbiol. 2019 Oct 22;19:230. doi: 10.1186/s12866-019-1602-8 (PMC6805388; doi:10.1186/s12866-019-1602-8)

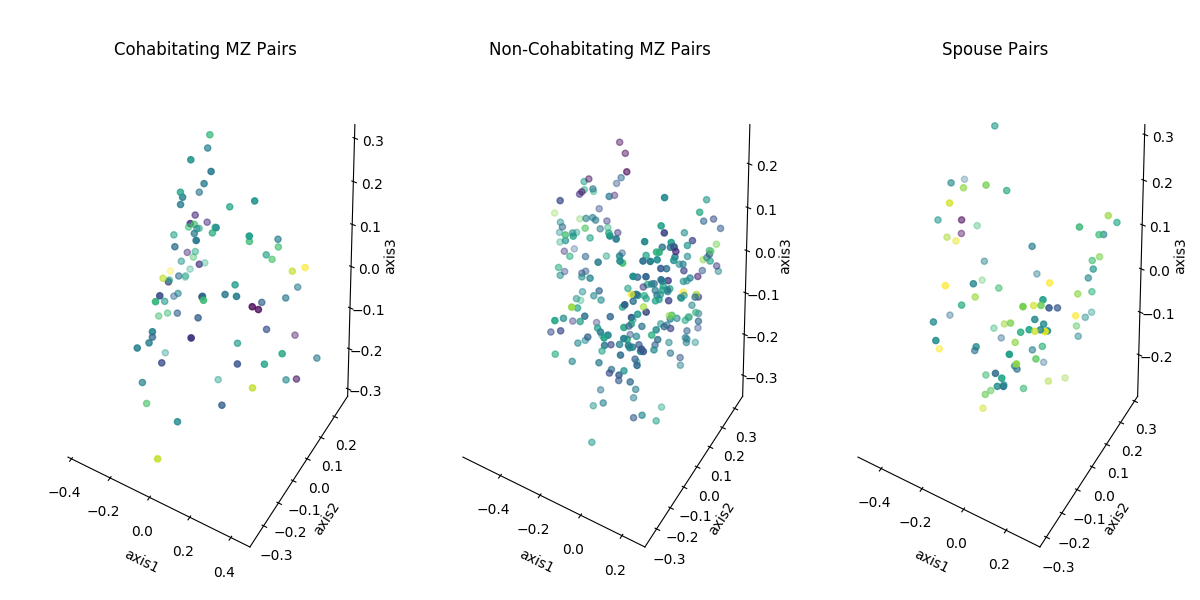

Supplement: Supplementary file 1 — Additional file 1: Fig. S1. PCoA plot generated from a Bray-Curtis dissimilarity matrix for visualization purposes. [file 12866_2019_1602_MOESM1_ESM.png]
